# Chromatin and oxygen sensing in the context of JmjC histone demethylases

**DOI:** 10.1042/BJ20140754

**Published:** 2014-08-22

**Authors:** Alena Shmakova, Michael Batie, Jimena Druker, Sonia Rocha

**Affiliations:** *Centre for Gene Regulation and Expression, MSI/WTB/JBC Complex, Dow Street, University of Dundee, Dundee DD1 5EH, Scotland, U.K.

**Keywords:** chromatin, chromatin remodeller, histone methylation, hypoxia, hypoxia-inducible factor (HIF), Jumonji C (JmjC), transcription, CD, chromodomain, CHD, chromodomain helicase DNA binding, CRC, chromatin-remodelling complex, FIH, factor inhibiting HIF, HIF, hypoxia-inducible factor, ISWI, imitation-SWI protein, JmjC, Jumonji C, KDM, lysine-specific demethylase, LSD, lysine-specific demethylase, NuRD, nucleosome-remodelling deacetylase, PHD, plant homeodomain, PHF, PHD finger protein, REST, repressor element 1-silencing transcription factor, VHL, von Hippel–Lindau protein

## Abstract

Responding appropriately to changes in oxygen availability is essential for multicellular organism survival. Molecularly, cells have evolved intricate gene expression programmes to handle this stressful condition. Although it is appreciated that gene expression is co-ordinated by changes in transcription and translation in hypoxia, much less is known about how chromatin changes allow for transcription to take place. The missing link between co-ordinating chromatin structure and the hypoxia-induced transcriptional programme could be in the form of a class of dioxygenases called JmjC (Jumonji C) enzymes, the majority of which are histone demethylases. In the present review, we will focus on the function of JmjC histone demethylases, and how these could act as oxygen sensors for chromatin in hypoxia. The current knowledge concerning the role of JmjC histone demethylases in the process of organism development and human disease will also be reviewed.

## INTRODUCTION

Changes to oxygen availability or increased oxygen demand create an imbalance called hypoxia. Hypoxia is an important physiological stimulus for embryo development of mammals, but it is also a serious component of the pathology of many human diseases [1–3]. Given its importance, it has attracted a great amount of research, which has helped to understand how the physiology of oxygen sensing and response works. However, at the cellular and molecular level, great unknowns still exist. The molecular mechanisms underlying the cellular response were significantly boosted by the discovery of the main transcription factor family controlled by oxygen, called HIF (hypoxia-inducible factor) in the early 1990s [4]. HIF is now known to be a heterodimer of the oxygen-controlled subunit HIF-α and the oxygen-insensitive subunit HIF-1β, which was originally identified as a binding partner for the aryl hydrocarbon receptor, and as such has the gene name of *ARNT* (aryl hydrocarbon nuclear translocator) [5].

In mammalians there are three genes for HIF-α subunits, HIF-1α, HIF-2α (gene name *EPAS1*, for endothelial Pas protein 1) and HIF-3α [6]. HIF-α is controlled by oxygen post-translationally via the action of dioxygenase enzymes such as prolyl hydroxylases and FIH (factor inhibiting HIF). Prolyl hydroxylase-mediated hydroxylation of HIF α-subunits creates a high-affinity binding site for the ubiquitin ligase complex containing the tumour suppressor VHL (von Hippel–Lindau protein) [7–10]. VHL is part of the cullin-2, elongin B/C and the small RING finger protein RBX1 ligase complex [11], which promotes Lys^48^-linked ubiquitination, and hence proteasomal-mediated degradation. FIH-mediated hydroxylation produces an inhibitory moiety in the C-terminal transactivation domain of HIF-1α and HIF-2α, preventing the binding of co-activator proteins such as p300 and hence reducing the transcriptional activity of the transcription factor [12].

HIF-dependent genes are varied and many, with over 100 direct genes being validated and several more putative genes recently identified by genomic approaches, such as ChIP-sequencing [13]. Genes involved in restoration of oxygen homoeostasis, cell survival and growth as well as metabolism have received great interest from the medical community, as these can be used not only as biomarkers, but also as direct therapeutic targets for diseases such as cancer [14].

One interesting new class of HIF-dependent targets are the dioxygenase enzymes called JmjC (Jumonji C)-containing proteins [15]. These enzymes are, for the most part, protein demethylases, and were identified as being structurally similar to the HIF hydroxylase FIH [16,17]. On the basis of the structural analysis of these enzymes, it became clear that these enzymes, much like FIH, would require molecular oxygen and 2-oxoglutarate to catalyse their enzymatic reactions, suggesting that JmjC enzymes could act as molecular oxygen sensors in the cell. In the present review, we will discuss the evidence available on how JmjC histone demethylases can contribute to oxygen sensing by altering chromatin structure and function.

## CHROMATIN AND HISTONE METHYLATION

Chromatin is the collective name for DNA and the protein complexes, of which the nucleosome (DNA and histone octamer) is the basic unit [18]. Initially thought as a passive impediment to nuclear processes, it is now known that chromatin is highly dynamic, and responsive to several stimuli and stages of development [19]. Two major states of chromatin are generally accepted: heterochromatin (compact/silent) and euchromatin (open/active) [18]. However, imaging approaches have revealed additional distinct compaction levels do exist in both types of chromatin domains [20].

**Figure 1:**
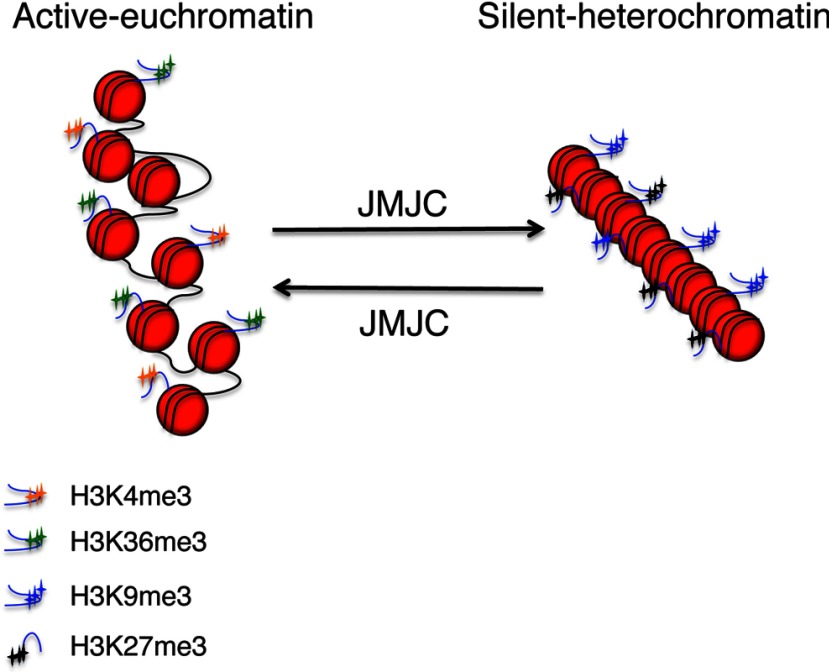
Histone methylation marks associate with chromatin compaction status Active chromatin or euchromatin is associated with a more open status and is characterized by several types of histone methylation marks as depicted. On the other hand, silent chromatin or heterochromatin is associated with closed and compacted status, also characterized by a subset of histone methylation patterns as depicted. JmjC enzymes can thus, in principle, control chromatin status by removing the histone methyl groups.

Chromatin structure can be altered by several mechanisms, either involving ATP-dependent remodellers [21] or changes to the histone octamer [22,23], including alternative histone and histone post-translation modifications. The number of possible histone modifications is vast; however, one such modification, with relevance to the present review, is histone methylation. Histone methylation can occur on several lysine or arginine residues, primarily on histone H3 and histone H4 [24]. Lysine methylation, unlike acetylation, does not change the charge of the protein. As such, methylation marks change chromatin structure by different mechanisms, involving the recruitment or inhibition of recruitment of distinct enzymatic complexes [25,26]. Functionally, histone methylation can both activate and repress transcription [25] as well as control DNA replication [26]. Histone methylation can lead to compaction or relaxation of chromatin (Figure 1), depending on which histone residue is methylated and, as such, how the recruitment of the corresponding enzymatic complex is altered [22].

Soon after its identification, histone methylation was thought to be a stable modification, as no enzymes were known to remove this mark. However, now it is known that histone methylation can be removed by two different classes of enzymes: the LSD (lysine-specific demethylase) family and the JmjC family (Table 1). For a more detailed review of the LSD family, please see [27].

**Table 1:** JMJC-containing protein in humans according to UniProt Y, yes; N, no.

Gene name	UniProt number	Additional names	Histone demethylase activity (Y/N)
*HR*	O43593	Hairless, HAIR	N
*HSPBAP1*	Q96EW2	HBAP1	N
*HIF1AN*	Q9NWT6	HIF1N, FIH	N
*JARID2*	Q92833	JARD2	N*
*JMJD1C*	Q15652	JHD2C	N†
*JMJD4*	Q9H9V9	-	N
*JMJD6*	Q6NYC1	PSR	Y‡
*JMJD7*	P0C870	-	N
*JMJD8*	Q96S16	-	N
*KDM2A*	Q9Y2K7	FBXL11, JHDM1A	Y
*KDM2B*	Q8NHM5	FBXL10, JHDM1B	Y
*KDM3A*	Q9Y4C1	JMJD1A	Y
*KDM3B*	Q7LBC6	JMJD1B	Y
*KDM4A*	O75164	JMJD2A	Y
*KDM4B*	O94953	JMJD2B	Y
*KDM4C*	Q9H3R0	JMJD2C	Y
*KDM4D*	Q6B0I6	JMJD2D	Y
*KDM4E*	B2RXH2	JMJD2E	Y
*KDM5A*	P29375	RBBP2, JARID1A	Y
*KDM5B*	Q9UGL1	PLU1, JARID1B	Y
*KDM5C*	P41229	JARID1C	Y
*KDM5D*	Q9BY66	JARID1D	Y
*KDM6A*	O15550	UTX	Y
*KDM6B*	O15054	JMJD3	Y
*JHDM1D*	Q6ZMT4	KDM7A	Y
*KDM8*	Q8N371	JMJD5	Y
*MINA*	Q8IUF8	MINA53, NO52	Y§
*NO66*	Q9H6W3	C14orf169	Y
*PHF2*	O75151	CENP35, JHDM1E	Y
*PHF8*	Q9UPP1	JHDM1F	Y
*TYW5*	A2RUC4	-	N
*UTY*	O14607	-	N

*No histone demethylase activity, but modulates methyltransferases.

†No evidence found yet.

‡Only *in vitro*.

§Not the main enzymatic activity.

## JmjC OXYGEN REGULATION

On the basis of the presence of the JmjC domain, there are currently 32 proteins in humans belonging to this class (Table 1). However, not all of these have been associated with histone demethylation. Although some of the JmjC proteins without histone demethylase activity have important biological functions in the cell, the present review will focus on the regulation of JmjC histone demethylases. As mentioned above, the JmjC class of proteins require oxygen and 2-oxoglutarate for their activity. Elegant structural work from the Schofield group and other laboratories has greatly helped in the understanding of how target selectivity is achieved, as well as with the general mechanism of demethylation by these enzymes [24,28–30].

For catalytic activity, JmjC histone demethylases use molecular oxygen and 2-oxoglutarate with release of CO_2_, succinate and formaldehyde [29,31]. As such, reduction in the availability of oxygen would have an impact on catalytic activity. Work performed using recombinant prolyl hydroxylases and FIH proteins has shown that these enzymes have different *K*_m_ values for oxygen [32]. Although the prolyl hydroxylase *K*_m_ value is approximately 230 μM O_2_, the FIH *K*_m_ value for O_2_ is only 90 μM [32]. These results indicated that prolyl hydroxylases are inhibited with a smaller reduction in the availability of O_2_ than FIH. However, thus far oxygen affinity for the majority of the JmjC enzymes has not been determined. One enzyme that has been investigated, which belongs to the KDM4 (lysine-specific demethylase 4) family, is KDM4E [33]. It was shown that KDM4E reacts slowly with O_2_, at a similar level to prolyl hydroxylase 2. It was also suggested that KDM4E has an incremental response over physiologically relevant ranges of O_2_ [33]. This analysis did indicate the potential of these enzymes to act as oxygen sensors in the cell. However, it would be necessary for more biochemical studies to be performed *in vitro* to really establish, and compare, the oxygen requirements of JmjC histone demethylases with other known dioxygenases such as prolyl hydroxylases and FIH.

An additional regulation of JmjC demethylases by oxygen, albeit indirect, is via transcriptional regulation. Transcriptional analyses, using several different cellular systems, have shown that a great number of JmjC histone demethylases are hypoxia- inducible at the mRNA levels [34]. These include KDM2A, KDM2B, KMD3A, KDM3B, KMD4B, KDM4C, KDM4D, KDM5A, KDM5A, KDM5B, KDM5C, KDM5D, KDM6A, KDM6B, KDM8, JARID2 and PHF8 (plant homeodomain finger protein 8) (references in [34]). Some of these enzymes have been shown to be direct targets of HIF-1α. The HIF-1α-regulated JmjC histone demethylases are: KDM3A [35–38], KDM4B [35,36], KDM4C [36], KDM5C [39] and KDM6B [40]. Whether any of the additional JmjC histone demethylases that were found to be hypoxia-inducible are also HIF-dependent remains to be investigated. In addition, their regulation by HIF-2 has not been directly investigated, apart from KDM3A, KDM4B and KDM4C, which are mainly regulated by HIF-1α [36]. As such, it is not known whether the remaining hypoxia-inducible JmjC enzymes are HIF-1-specific targets. Current studies should help elucidate these questions.

## JmjC ACTIONS ON CHROMATIN STRUCTURE AND CHROMATIN REMODELLERS

Histone methylation is probably one of the most studied chromatin modifications with a great number of studies describing the role of a specific methylation mark in the control of gene expression. The recent availability of large-scale and genomic sequencing data has also helped to associate different methylation marks with the diverse chromatin states [41]. Generally, methylation at Lys^4^, Lys^36^ or Lys^79^ of histone H3 are hallmarks of actively transcribed genes, whereas methylation of Lys^9^ and Lys^27^ of histone H3, as well as of histone H4 Lys^20^, are associated with transcriptional repression and heterochromatin formation [42] (Table 2). Mutual exclusiveness of these marks establishes the importance of histone demethylases in the remodelling of chromatin and reprogramming of gene expression.

**Table 2:** Methylation marks as a determinant of chromatin states

Methylation mark	Associated activity
H3K4me2/3	Hallmark of regulatory elements at the 5′ end of transcriptionally active genes or of genes poised for transcriptional activation
H3K4me1	Hallmark of enhancer sequences
H3K36me1/2	Restricted to the body and 3′ end of the gene
H3K36me2/3 and H3K79me2/3	H3K36me2/3 and H3K79me2/3 are enriched in gene bodies
H3K9me3 and H4K20me3	Associate with non-genic regions, repetitive or transposable DNA elements including satellite sequences and long terminal repeats
H3K4me3 and H3K27me3	Bivalent domain in embryonic stem cells associated with complex transcription

Considering the role of the methylation marks and demethylase specificity, KDM2 and KDM5 families can promote the formation of repressed chromatin, KDM3, KDM6 and KDM7 act as chromatin activators and the KDM4 family may have different effects on chromatin status [41]. However, less is known about the interplay between histone demethylases and other important types of chromatin-remodelling enzymes, such as the ATP-dependent chromatin remodellers [CRCs (chromatin-remodelling complexes)].

**Figure 2:**
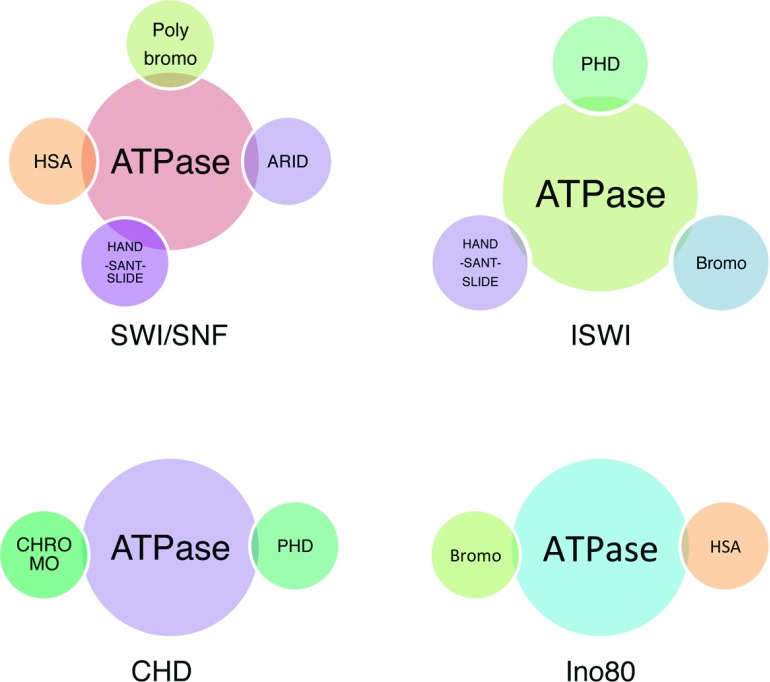
Schematic representation of chromatin-remodelling complexes, highlighting the domains present in different subunits ARID, AT-rich interactive domain; HAND-SANT-SLIDE, DNA-binding domains; HSA, domain binding nuclear actin-related proteins (ARPs) and actin; NuRF, ISWI-related chromatin remodeller.

Four subfamilies of CRCs have been characterized in mammals: SWI/SNF, CHD (chromodomain helicase DNA binding), Ino80 and ISWI (imitation-SWI protein) [34]. They share a similar ATPase domain responsible for the disruption of protein–DNA interactions in nucleosome using the energy of ATP hydrolysis. However, all four subfamilies specialize in particular purposes and biological context, depending on their interaction network [18,21]. The broad interaction network of chromatin remodellers is regulated by unique domains flanking the ATPase domain or by the presence of accessory subunits (Figure 2).

There is very little information concerning how JmjC enzymes interact or control the action of CRCs. However, a few studies have suggested that demethylases can alter the action of such remodellers. LSD1 and several JmjC enzymes were found in a genetic screen in *Drosophila* as corepressors of SWI/SNF activity during wing development [43]. In addition, KDM6A was shown to interact with BRM, one of the catalytic helicases in the SWI/SNF complex, modulating the acetylation of H3K27 (where K indicates the lysine residue under investigation, i.e. H3K27 is Lys^27^ of histone H3) via binding with CBP (cAMP-response-element-binding protein-binding protein) [44].

**Figure 3:**
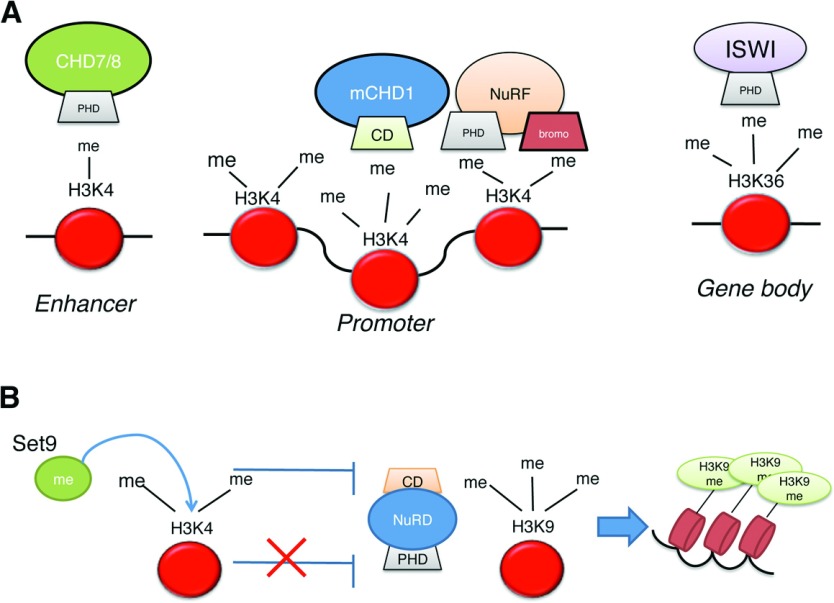
How histone methylation controls CRC recruitment (**A**) Histone methylation marks localization and chromatin remodellers associated with them. bromo, bromodomain; me, methylation site. (**B**) Regulation of NuRD recruitment by histone methylation marks. Set9, H3K9 methyltransferase.

Despite the lack of direct research investigating how JmjC control the action of CRCs, indirect evidence does exist to support this hypothesis. As such, ISWI- and CHD- remodelling complexes contain tandem CDs (chromodomains) and PHDs (plant homeodomains) that are able to recognize methylated lysine residues (Figure 3A). Histone methylation was shown to be important for the recruitment and stabilization of CRCs [45–47]. Different methylation marks are associated with ISWI and CHD binding. They are recruited to promoter and enhancer elements enriched in H3K4 methylation, known to regulate transcription initiation [45–47], recruited to methylated H3K36 in gene bodies linking remodellers to transcription elongation and termination [48] and recruited to the H3K9 methylated regions of repressed chromatin (Figure 3A). As such, given the importance of histone methylation for CRC recruitment, it would be rational to hypothesize that histone demethylases can regulate this process. Future research directed at this particular question would be necessary to fully establish how JmjC enzymes co-ordinate the action of CRCs.

An interesting example of the action of CRCs controlled by histone methylation marks is the recognition of methylated lysine residues by the NuRD (nucleosome-remodelling deacetylase) complex (Figure 3B). NuRD is a repression complex that combines deacetylase activity with ATP-remodelling activity to form a compacted chromatin state, and thus repress gene expression. It contains a CHD3/CHD4 ATPase domain with tandem PHDs and CDs in its N-terminus. The PHD fingers were shown to interact with H3K9me3 [where me indicates the methylation status, from me1 (monomethylated) to me3 (trimethylated)] and this interaction can be regulated by the methylation status of H3K4 [49]. Methylation of H3K4 by Set9 reduces the association of the complex with the histone H3 tail, which is the most probable mechanism for regulating the formation of repressed chromatin by the NuRD complex [50]. The histone demethylase LSD1 has been linked with the NuRD complex [51], indicating that indeed histone demethylases can modulate CRC's activity. However, the involvement of JmjC in the control of NuRD recruitment has not been investigated thus far.

Almost all CRCs apart from CHD have been implicated in the hypoxia response [52–55]. As such, and considering the level of cross-talk present in chromatin remodelling, more links between lysine demethylation and ATP-dependent chromatin remodelling will be discovered in the near future.

## JmjC HISTONE DEMETHYLASES AND TARGET SPECIFICITY IN LOW OXYGEN

**Figure 4:**
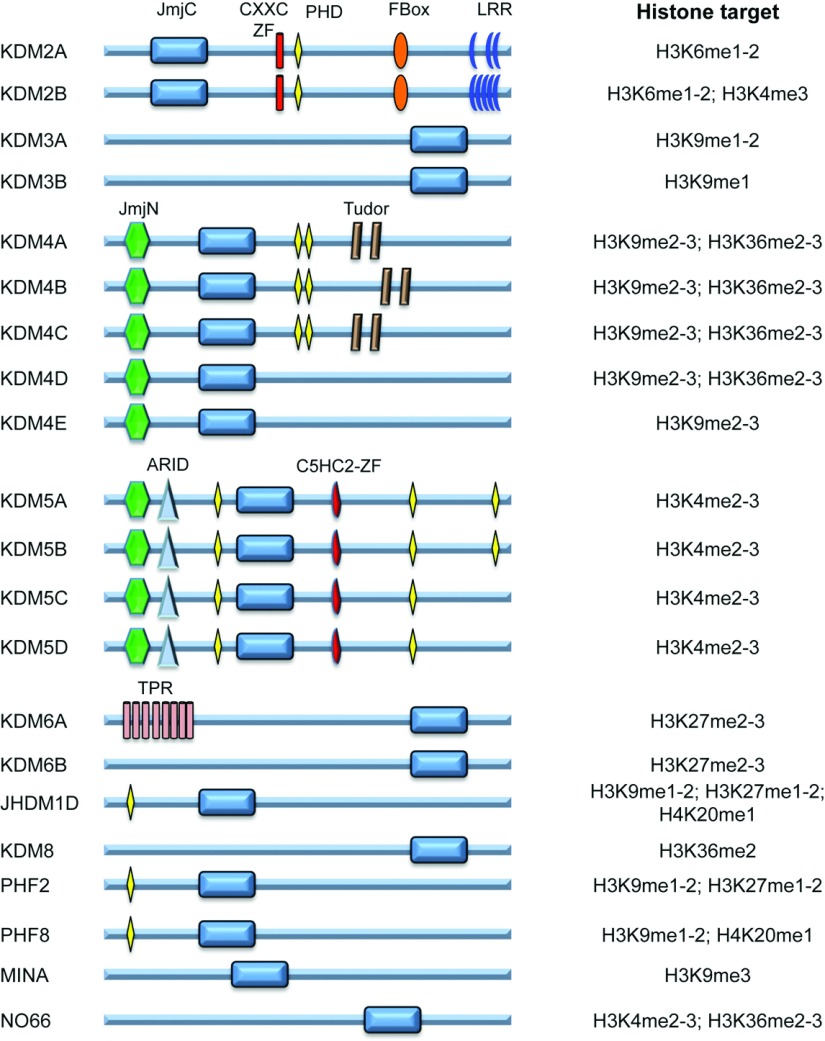
JmjC family domain structure and histone targets ARID, AT-rich interactive domain; C5HC2-ZF, C5HC2 zinc finger domain; CXXC-ZF, CXXC zinc finger domain; FBox, F-box domain; JmjC, Jumonji C domain; JmjN, Jumonji N domain; LRR, leucine-rich repeat domain; TPR, tetratricopeptide domain; Tudor, Tudor domain.

KDM2A was the first published JmjC-domain-containing protein shown to have histone demethylase activity [31]. Since then, many more JmjC histone demethylases have been uncovered and their histone targets investigated. *In vitro* studies have revealed that while some JmjC have quite particular selectivity for histone residues, others have a broader range of targets (Figure 4). However, which JmjC histone demethylase is active in cells is still unknown, and most likely will vary from cell type to cell type, as well as developmental state.

**Figure 5:**
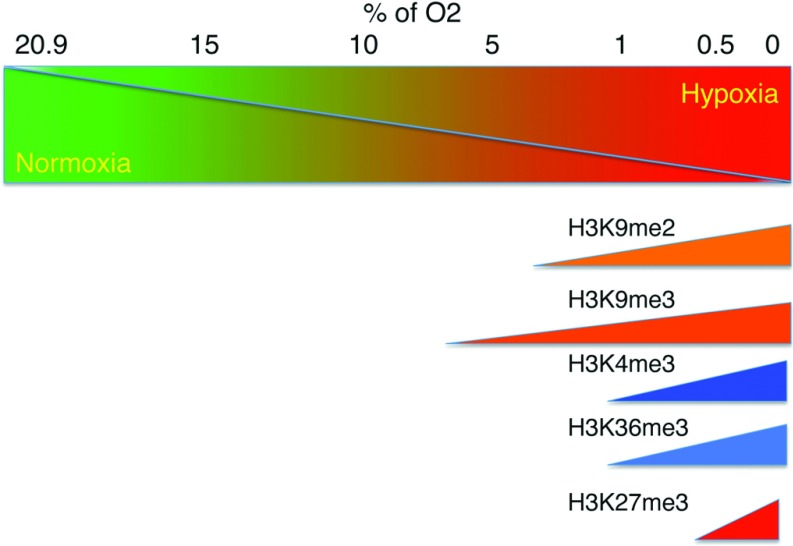
Hypoxia induces the increase of certain histone methylation marks Diagram depicting the relationship between oxygen concentration and increased levels of histone methylation marks observed in several studies.

Studies in hypoxia have shown that histone methylation marks are indeed increased (Figure 5) [56–58]. Exposure of Hepa1-6 cells to 0.2% O_2_ for 48 h induces global increases in H3K4me2, H3K4me3, H3K79me3, H3K27me3 and H3K9me2 [56]. Another study found that changes in histone methylation marks in mouse macrophage cells were only visible after 24 h of exposure when oxygen levels were below 3%. Furthermore, at 1% oxygen there was a global increase in H3K9me2, H3K9me3 and H3K36me3, and these changes were attributed to inhibition of histone demethylase activity [58]. These studies suggest that a variety of JmjC enzymes can be inhibited by low oxygen conditions; however, given that most of these enzymes can target several histone residues (Figure 4), additional work is required to really establish which enzymes are altered in hypoxia.

One study has reported a global increase in H3K4me3 at 1% oxygen, as a result of KDM5A inhibition [57]. That study also observed an increase in H3K4me3 at *HMOX1* (haem oxygenase 1) and *DAF* gene promoters [57], thus suggesting that global and local increases in H3K4me3 due to KDM5A inhibition during hypoxia may lead to altered gene transcription.

However, there is still very little information about the role of histone methylation on chromatin structure and JmjC-specific actions during hypoxia. One important question to answer is how are particular histone modifications associated with active and repressive transcription altered following hypoxic stress. In addition, dynamic analysis is also lacking, as the oxygen sensing and response system in cells is usually programmed to reset after prolonged hypoxia, as is observed in the regulation of HIF levels by prolyl hydroxylases. As such, it would be hypothesized that changes in histone methylation marks would be more dramatic and directly dependent on JmjC inhibition at earlier times of hypoxia exposure. Prolonged hypoxia would involve not only inhibition of these enzymes, but also, and most likely, compensatory mechanisms by which increased expression of JmjC proteins would occur.

## JmjC FUNCTIONS IN DEVELOPMENT

Although cell culture studies have revealed important information on the complex molecular functions, regulation and targets of JmjC histone demethylases, whole organism studies are vital in understanding the biological significance of these enzymes *in vivo*. The importance of JmjC demethylases in biological processes is illustrated by their association with diseases and depletion studies in model organisms (Table 3), showing that they have key roles in development. Studies in invertebrate model organisms found developmental defects when certain JmjC enzymes are depleted. For example, knockout of two PHF family orthologues in zebrafish, Jhdm1da and Jhdm1db, leads to abnormal posterior development [59]. Also knockdown of Lid, the KDM5 family orthologue in *Drosophila*, causes numerous developmental defects through deregulation of homeotic gene expression [60].

**Table 3:** Phenotypes associated with JmjC depletion studies in model organisms –, no orthologue; ND, not determined or no data.

	Phenotype in depletion studies			
JmjC	*Mus musculus*	*Danio rerio*	*Caenorhabditis elegans*	*Drosophila melanogaster*
KDM2B	Partial peri/postnatal lethal, lowered sperm count, defective neural tube development [112]	ND	ND	Embryonic lethal [113]
KDM3A	Metabolic defects, adult obesity and male infertility [114–116]	–	–	ND
KDM4A	Impaired cardiac stress response [60]	ND	Increased germline apoptosis [117]	Abnormal male wing extension and reduced male lifespan [118]
KDM4D	No detected phonotypical change [119]	ND		
KDM5A	Decreased apoptosis in HSC and myeloid progenitors [120]	ND	Defects in vulva formation and reduced lifespan [83,121]	Developmental defects [122]
KDM5B	Embryonic lethal [123]	ND		
KDM5C	Cardiac looping and neuralation defects [124]	Increased neuronal cell death and abnormal dendrite development [90]		
JARID2	Embryonic lethal [125–127]	ND	–	ND
KDM6B	Perinatal lethal and defective lung development [128]	ND	Defective vulva development [129]	Larval lethal [130]
KDM6A	Partial male embryonic lethal, defects in neural tube and cardiac development and female embryonic lethal [63,124]	Abnormal posterior development [59]		
PHF2	Partial neonatal lethal, adipogenesis defects [61]	Detects in brain development [131]	Defects in body movement [97]	–
KDM8	Embryonic lethal [132]	ND	ND	ND
JMJD6	Perinatal lethal with multiple developmental defects [133]	Defects in heart brain, somites and notochord [134]	Mild cell engulfment defects [135]	Enhanced apoptosis in developing eye [136]

Several JmjC histone demethylases have been depleted in mice (Table 3). Phenotypes of these knockouts have large variation, ranging from embryonic lethality in KDM8, JARID2 and KDM5B knockouts to male infertility and obesity in the KDM3A knockout, to no detectable phenotypical change in KDM4D depletion. This demonstrates that, although some of these demethylases are essential in development, functional redundancy partly as a result of overlapping targets is likely to account for milder phenotypes observed from depletion of others. Mechanistic insights have been gained from some of these studies. For example, PHF2-knockout mice have reduced adipose tissue, PHF2 interacts with the key regulator of adipogenesis CEBPA and is proposed to promote adipogenesis through co-activation of CEBPA [61,62]. This has led to the suggestion that PHF2 may be a novel therapeutic target for the treatment of obesity and metabolic diseases.

The cardiac defects observed in KDM6A-depleted mice are likely to be due to deregulation of cardiac gene expression programmes, as KDM6A is recruited to cardiac-specific enhancer regions where it associates with multiple transcription factors [63]. However, for several of the depletion studies performed, molecular mechanisms to explain the observed phenotypes are still ill-defined and will require further investigation. Furthermore, for many of the JmjCs, there is limited information from *in vitro* studies and no/limited information from *in vivo* studies, which will need to be addressed in order to characterize these enzymes.

## JmjC FUNCTIONS IN HUMAN DISEASE

**Figure 6:**
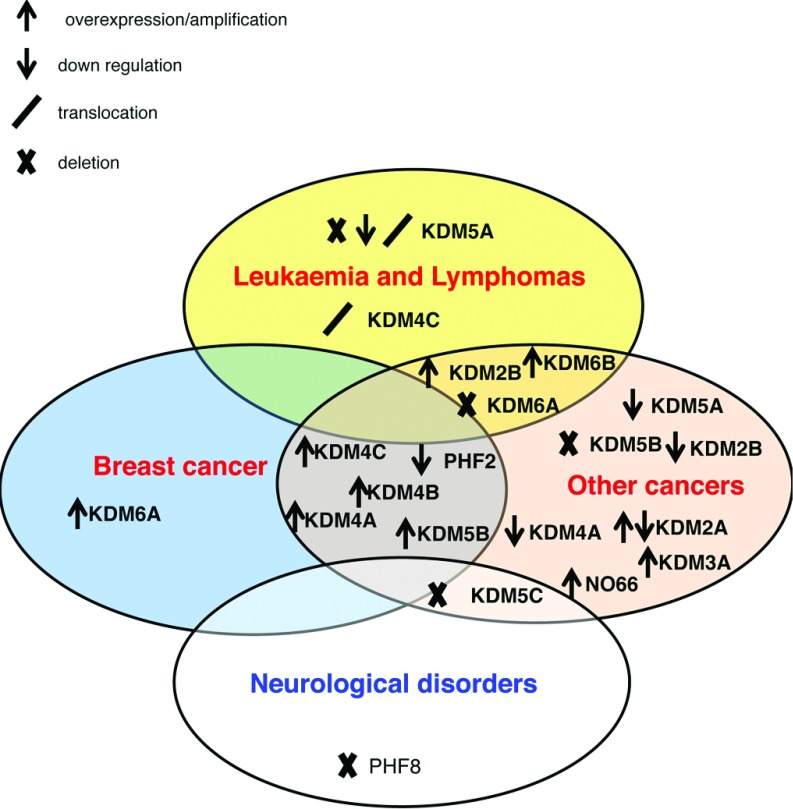
JmjC alterations in human disease Diagram of the observed associations of JmjC expression in different human diseases, highlighting KDM4 as particularly susceptible to deregulation in a variety of human pathologies.

One aspect that highlights the importance of a particular gene is its association with human disease. As such, there are numerous links between JmjC histone demethylases and human diseases, most notably cancer and neurological disorders (Figure 6). Genetic alterations in JmjCs, including KDM2A, KDM2B, KDM4A, KDM4B, KDM42C and KDM5B, have all been linked to cancer progression and these enzymes have been suggested as chemotherapeutic targets [64–73].

The KDM2 group of JmjCs appear to have both cancer-promoting and -inhibiting functions depending on cellular context, through their regulation of cell proliferation. In cell culture, NF-κB (nuclear factor κB)-dependent colon cancer cell growth is impaired by KDM2A [74] and KDM2B has been demonstrated to negatively regulate cell proliferation via repression of ribosomal RNA genes [75]. Conversely, overexpression of KDM2A and KDM2B in mouse embryo fibroblasts confers resistance to stress-induced senescence [66]. KDM2B positively regulates cell proliferation and growth through silencing of the cell cycle inhibitor p15^Ink4b^ [66,76]. KDM2A is overexpressed in a subset of non-small cell lung cancer patients and has been reported to drive cancer progression in these tumours through up-regulation of ERK1/2 (extracellular-signal-regulated kinase 1/2) signalling [64]. KDM2B has been identified both as oncogene and tumour suppressor through proviral insertional mutagenesis studies in rodents [77,78]. Various leukaemias and bladder cancers display up-regulated KDM2B [65,66] and it is proposed to play a key role in leukaemia progression through loss of p15^Ink4b^ function [65]. As well as being overexpressed in certain cancers, KDM2A and KDM2B have also been found to be down-regulated in prostate cancer and glioblastoma respectively [75,79]. These studies have highlighted the cell-dependent nature of the KDM2 family's mode of action.

The KDM4 group has also been associated with various cancers. KDM4A is down-regulated in bladder cancer [80], and KDM4A and KDM4B are up-regulated in breast cancer and peripheral nerve sheath tumours respectively [67,81]. KDM4B is oestrogen inducible and has been shown to promote oestrogen-stimulated breast cancer proliferation [82]. Amplification of KDM4C has been reported in breast cancer, oesophageal squamous cell cancer and medulloblastoma [68,69,83] and a translocation involving KDM4C is present in mucosa-associated lymphoid tissue lymphomas [70]. KDM4C overexpression induces transformation in immortalized mammalian epithelial cell lines, suggesting a role for KDM4C in driving tumorigenesis [83]. This is supported by studies showing that knockdown of KDM4C limits proliferation in mammalian cancer cell lines [83–85].

The KDM5 group also has strong association with human diseases. KMD5A is associated with tumourigenesis in the lung [86] and is also associated with haemopoietic malignancies [87]. KDM5B has low expression levels in normal adult tissue, except in the testes, but it is found overexpressed in the bladder, prostate and breast cancer [71–73]. KDM5B is a transcriptional co-activator of the androgen receptor, but can also function as a transcriptional repressor [88]. It has also been shown to promote breast cancer proliferation in both *in vitro* and *in vivo.* Depletion of KDM5B limits growth in MCF-7 breast cancer cells and in a mouse breast cancer model, and this correlates with repression of tumour suppressor genes including BRCA1 (breast cancer early-onset 1) [89].

Several JmjCs, including KDM5C and PHF8, have strong links to neurological development and defects. KDM5C is important for neuronal survival in primary mammalian neurons and dendritic development in zebrafish [90]. Furthermore, mutations in KDM5C are frequently found in X-linked mental retardation [91–94]. KDM5C is involved in REST (repressor element 1-silencing transcription factor)-mediated transcriptional repression and loss of KDM5C activity, leading to deregulation of neuronal genes under transcriptional regulation of REST, which has been proposed as the mechanism between KDM5C and X-linked mental retardation [95]. PHF8 loss-of-function mutations have also been found in X-linked mental retardation patients as well as patients with cleft palate [96], it is suggested that KDM5C and other X-linked mental retardation-associated genes are under regulation by PHF8 via the transcription factor ZNF711 (zinc finger protein 711) [97].

KDM6A has been associated both with cancer and X-linked syndromes such as Kabuki syndrome [98–101]. Similarly, KMD6B contributes to tumour suppression by activation of p14^ARF^ [102], as well as co-operating with p53 [103]. It has also been associated with pro-inflammatory gene expression in immune cells [104–106].

## CONCLUSIONS

Epigenetic deregulation is a key driver of tumorigenesis and neurological disorders as well as other diseases. The pathological roles of enzymes modifying histone methylation, including JmjC histone demethylases, is only beginning to be understood and these enzymes provide promising drug targets. As such, several studies have already attempted to employ structural and medicinal chemical strategies to target these enzymes for therapy [104,107–111]. As JmjC histone demethylases require oxygen as a cofactor, it is possible to speculate that histone marks can be rapidly altered when hypoxia is present. This would indicate that chromatin structure would change and adapt to low oxygen, possibly even more rapidly than any other process in the cell, and would thus set the landscape for the hypoxia-induced transcriptional program observed in many cells. However, additional work regarding the specific requirements for oxygen for each of the JmjCs is required to fully verify this hypothesis. As more information is gathered regarding the molecular function of the individual JmjC enzymes, more targeted approaches can be employed. Thus there is still great potential for using JmjC enzymes as valid new targets for therapy in human disease. Future research analysing the role of individual JmjC enzymes, and their relationship with chromatin-remodelling complexes, should provide exciting and useful insights into the biology of JmjC enzymes as well as exploit their potential for therapeutic targeting.
